# Supplementary material for: Validity of ICD codes to identify do-not-resuscitate orders among older adults with heart failure: A single center study
Source: PLoS One. 2023 Mar 13;18(3):e0283045. doi: 10.1371/journal.pone.0283045 (PMC10010557; doi:10.1371/journal.pone.0283045)
Supplement: Attachment — Submitted filename: callahan DNR review.docx [file pone.0283045.s001.docx]

Dear PLOS One Staff

Thank you for allowing me to review the manuscript entitled “Validity of ICD Codes to Identify Do-Not-Resuscitate Orders” by Callahan et al. I commend the authors on investigating an important topic. DNR-status is an important patient-descriptor, is strongly associated with hospital mortality, and can be a source of significant confounding in comparative effectiveness and observational research. The ability to identify DNR status in administrative discharge and claims databases would be of great benefit to health services research. While the primary objectives of the study are commendable, I have several concerns about the execution of the study. My concerns are primarily related to cohort selection, potential biases in interpretation related to the choice of patients with heart failure, potential biases from a single center study, and the lack of racial/ethnic diversity in the cohort. Many of the concerns are likely not addressable but these concerns should temper the authors conclusions. I believe the study does have merit but is in need of substantial modifications and revisions. I have outlined my concerns in a list of both Major and Minor points below. Please feel free to contact me should you have further questions.

Sincerely,

Anuj Mehta, MD MS

**Major Points**

1. My primary concern regarding the manuscript is the disconnect between the generalizability with which the authors discuss their results and the restrictive nature of their cohort. The authors use their results to discuss how discharge and claims data can be used to identify DNR orders in future studies. However, their cohort is exceedingly restrictive and may not be the most appropriate with which to identify patients admitted with heart failure. Their reported statistics of sensitivity, specificity, PPV, and NPV are only valid for this specific cohort and likely are not generalizable to other conditions or patient cohorts. The authors should first consider their patient selection along the below delineated lines and also discuss their results with much greater clarity about the limitations of their findings throughout the abstract and manuscript, not just in the limitations section of the discussion.
   a. Age – the authors select patients who are ≥65 years of age. Use of DNR orders does increase with age meaning there would be greater prevalence of DNR orders in an older cohort. However, previous studies have indicated use of DNR orders begins to increase at around age 40. Use of an older cohort would result in higher sensitivity and specificity due to the higher prevalence. The authors should address this by either widening the cohort or doing a sensitivity analysis which includes patients down to age 40.
   b. Cohort definition – the authors only include patients with a principle diagnosis of HF (ICD9 and ICD10) as the ‘HF’ cohort. However, previous work (Mehta et al. Journal of Critical Care. 2015.) demonstrates that the most severely ill patients with HF (i.e. those most likely to have a DNR order) may have a principle diagnosis of acute respiratory failure and a secondary diagnosis of HF (i.e. presented with hypoxia due to pulmonary edema) or a principle diagnosis of acute MI with a secondary diagnosis of HF. These alterative coding pathways may be used to increase reimbursement but still tend to identify patients with HF. The authors could consider a sensitivity analysis with a wider cohort definition or they should mention that their definition may have selectively excluded individuals most likely to have a DNR order.
   c. Choice of heart failure – the authors restrict their analysis to HF only for unclear reasons. They suggest that studies that include all patients may include diagnoses for which DNR may not be relevant. However, DNR orders issued early in the course of admission tend to be more indicative of comorbid condition rather than the acute presenting illness. Moreover, other cohorts such as cancer may be even more relevant for DNR orders. The authors should provide greater justification for why HF was chosen, what biases such a choice may create, and limit their discussion about the utility of their findings to patients with HF.
2. My second major concern deals with the use of a single center and conclusion that can be drawn from it. I commend the authors on their sample size and efforts but I am concerned about any conclusions drawn from a single center on generalizable billing practices that might be heavily biased. The authors correctly mention several possible reasons why DNR orders may be systematically over or under estimated in billing codes from a given center. Each health center tends to bill in a unique fashion. Biases in terms of including or not including certain codes tend to happen at the hospital level, not the patient level. Therefore, the use of a single health system does not necessarily reduce the potential of coding bias to either over or under represent DNR orders in ICD codes. The authors should be far more specific in addressing the limitations of a single center in their discussion. Specifically, they should address the potential for different coding practices at different hospitals to result in significantly different results that what they have observed.
3. I am also concerned about the lack of racial diversity in this single center study. There is extensive evidence indicating that different racial groups have significantly different rates of ACPs and DNR orders when inpatient. Specifically, patients who identify as black are far less likely to have a DNR order when hospitalized. Therefore, a population with greater racial diversity may have very different performance metrics for iDNR vs eDNR. The authors should address this issue in the discussion.
4. The authors use and presentation of the CCI is confusing. The CCI is a continuous score ranging from 0 to 37 based on a weighted assessment of each individual comorbidity. The authors indicate that they are using the CCI but then present data in categories of 0, 1, 2-3, and 4+. The language used in the results seems to indicate that these are not categories of the scores but rather number of comorbidities. As each comorbidity carries different weights, simply counting the number of comorbidities is misguided. Moreover, when considering advanced directives, certain comorbidities are far more associated with a DNR order (e.g. cancer) than others. Therefore, simply looking at the CCI to compare differences between the iDNR and eDNR group can lead to misguided conclusions. I would suggest the authors clarify how the CCI is being used in the models and would present different rates of certain key comorbidities like cancer.
5. I am additionally confused by the use of cost as a potential outcome. The fact that patients with a DNR order have lower costs is not surprising or informative. Patients with a DNR order are less likely to receive high cost invasive therapies and, given associations with mortality, have shorter length of stays. Moreover, I am also confused by the primary exposure in the cost analysis as presented in Table 4. It appears that the primary exposure is a 4 level categorical variable with levels of Died, DNR, Died & DNR, and Lived & no DNR. This variable is very confusing as there are potentially overlapping categories and potentially groups not represented. For example, if a patient died but had no DNR does that mean they are level 1 or is there overlap between level 1 and level 3. Moreover, if the groups are mutually exclusive, is level 2 really Lived and DNR? The authors need to clarify why cost is an important outcome and how they are defining the exposure. One might consider looking at LOS rather than cost given that costs can vary widely between hospitals.
6. Several studies have shown significant changes to coding patterns associated with the shift from ICD-9 to ICD-10 in October 2015.While the authors address the validity of iDNR in several subgroups, they do not report differences in iDNR validity for ICD-9 vs ICD-10 codes. I would strongly suggest the authors do further subgroup analysis focusing on ICD-9 vs ICD-10 to show that secular changes in coding pratices and the shift from ICD-9 to ICD-10 has not changed validity.

**Minor Points**

1. In the introduction, the authors indicate that DNR is a legal document. However, in most states it is a hospital order that only applies to that hospitalization. Medical Orders for Life Sustaining Treatments (MOLST) forms have taken over in many states as the primary method of communicating goals of care between hospitalizations. In most states, MOLST forms are legal documents. The authors should just clarify this point about DNR orders.
2. The Methods Data Source section states that ‘both authors had full access to all the data’. There are 3 authors so I would suggest modifying the text to indicate whether all others, 2/3 authors, etc had access to the data.
3. In the results discussing mortality, the section around non-DNR drivers of mortality is a bit distracting. Knowing that “other pay sources” had the highest mortality is not related to the central question of iDNR vs eDNR.
4. Figure 1 is really a Table, not a figure.
5. Previous work has shown that DNR status can be a major confounder in comparative effectiveness and observational research (Bradford et al. CCM. 2014). Being able to identify DNR status in large administrative databases would enable to researchers to adjust for this key confounder if/when necessary. The authors should explore this fact in the discussion.
